# Diagnostic value of GPX4, IL-13, periostin, and thiol/disulfide balance in adult patients with scorpion envenomation: a prospective observational study

**DOI:** 10.3389/ftox.2025.1697677

**Published:** 2025-11-06

**Authors:** Hasan Buyukaslan, Şenay Koçakoğlu

**Affiliations:** 1 Department of Emergency Medicine, Faculty of Medicine, Harran University, Şanlıurfa, Türkiye; 2 Department of Family Medicine, Faculty of Medicine, Harran University, Şanlıurfa, Türkiye

**Keywords:** biomarkers, emergency medicine, GPx4, IL-13, oxidative stress, periostin, scorpionsting, thiol/disulfide balance

## Abstract

**Background and Objectives:**

Scorpion envenomation presents with a wide spectrum of clinical manifestations, ranging from mild local symptoms to severe systemic complications. This study aimed to evaluate the diagnostic and prognostic utility of GPX4, IL-13, periostin, SDF-4, and thiol/disulfide homeostasis parameters in adult patients with confirmed scorpion envenomation.

**Materials and Methods:**

This prospective observational study included 60 adult patients with confirmed scorpion stings and 33 healthy controls. Serum levels of GPX4, IL-13, periostin, and SDF-4 were measured using ELISA. Thiol/disulfide balance was evaluated by spectrophotometric assay. Clinical severity was graded using a four-level scale (Grade I–IV) based on local and systemic findings. Receiver operating characteristic (ROC) analysis and logistic regression were performed to assess the diagnostic performance and independent predictive value of biomarkers.

**Results:**

Patients exhibited significantly lower GPX4, native thiol, and total thiol levels, and higher disulfide, IL-13, periostin, and SDF-4 levels compared to controls (p < 0.001). The disulfide/native thiol and disulfide/total thiol ratios were also elevated. GPX4 (AUC = 0.984), SDF-4 (AUC = 0.900), and periostin (AUC = 0.850) demonstrated excellent diagnostic accuracy. GPX4 and disulfide levels were identified as independent predictors of envenomation. Biomarker levels significantly correlated with clinical severity grades.

**Conclusion:**

Oxidative and inflammatory biomarkers, particularly GPX4, disulfide/native thiol ratio, IL-13, and periostin, provide diagnostic and prognostic value in scorpion envenomation. Incorporating these parameters into clinical assessment may enhance early risk stratification and guide management in the emergency setting.

## 1 Introduction

Scorpion envenomation remains a serious public health issue in many parts of the world, with more than one million cases and an estimated 3,000 deaths reported annually (Chippaux and Goyffon, 2008). Although over 1,500 scorpion species have been identified, only around 30 have venoms that are clinically well-characterized (Possani et al., 1999; Isbister and Bawaskar, 2014). The majority of severe cases occur in regions such as South and Central America, North Africa, the Middle East, and South Asia (Isbister and Bawaskar, 2014; Bosnak et al., 2009). In Türkiye, particularly in the Southeastern Anatolia Region, venomous scorpion species are frequently encountered.

The pathophysiological mechanisms underlying the transition from local envenomation to systemic toxicity are complex and not yet fully understood. Scorpion venom contains neurotoxins that stimulate the autonomic nervous system and provoke the release of inflammatory cytokines and reactive oxygen species (ROS), contributing to systemic inflammatory response syndrome (SIRS) and multiorgan dysfunction (Isbister and Bawaskar, 2014; Daachi et al., 2020). Oxidative stress resulting from an imbalance between ROS production and antioxidant defense plays a crucial role in venom-induced tissue injury. Thiol/disulfide homeostasis is a sensitive indicator of redox balance; alterations in this system have been documented in various acute inflammatory states but remain underexplored in scorpion envenomation, particularly in adults (Erel and Neselioglu, 2014; Aydin et al., 2020). Currently, the diagnosis and severity assessment of scorpion envenomation rely primarily on clinical observation, which can be subjective and delayed. There is a lack of rapid, objective biomarkers that can accurately predict disease progression or guide therapeutic decisions in the early phase of envenomation.

Glutathione peroxidase 4 (GPX4), a selenoprotein, protects cellular membranes against lipid peroxidation and suppresses ferroptosis, an iron-dependent form of oxidative cell death (Friedmann Angeli et al., 2014; Hu et al., 2025). Periostin, a matricellular glycoprotein induced by IL-13 and IL-4, is implicated in tissue remodeling and fibrosis and serves as a biomarker of type 2 immunity-related inflammation (Masuoka et al., 2012; Conway et al., 2014; Wynn, 2003). Similarly, IL-13 has been shown to play a central role in mucosal inflammation and fibrotic responses (Donlan et al., 2021; Sogabe et al., 1998). Although these molecules have been widely studied in allergic and fibrotic conditions, their roles in scorpion envenomation remain largely unknown.

Stromal cell-derived factor 4 (SDF-4), also known as Cab45, is a calcium-binding protein involved in Golgi function, protein trafficking, and stress signaling, with potential as a novel biomarker in inflammatory disorders (Sogabe et al., 1998; Kudo-Saito et al., 2021). The diagnostic relevance of SDF-4, GPX4, IL-13, periostin, and thiol/disulfide balance in scorpion envenomation has not been systematically investigated.

This study aims to evaluate the diagnostic utility of IL-13, periostin, GPX4, SDF-4, and thiol/disulfide homeostasis parameters in patients with scorpion envenomation. Identifying early biochemical alterations in these markers may offer valuable insights into the underlying pathophysiology and improve diagnostic precision in clinical practice.

## 2 Materials and methods

### 2.1 Ethics

The study was approved by the Harran University Clinical Research Ethics Committee (Approval No: HRÜ/25.07.51, Date: 14 April 2025). All procedures were conducted in accordance with the principles of the Declaration of Helsinki. Written informed consent was obtained from all participants or their legal representatives prior to inclusion.

### 2.2 Study design and participants

This prospective observational study was conducted at Harran University Faculty of Medicine, Department of Emergency Medicine between 15 April 2025 and 23 August 2025. Patients presenting with scorpion sting envenomation were prospectively enrolled. A total of 60 adult patients with confirmed scorpion stings formed the envenomation group, and 33 age- and sex-matched healthy individuals without any history of scorpion sting constituted the control group.

Inclusion criteria: The inclusion criteria were defined as follows: for the patient group, a confirmed scorpion sting based on clinical findings, patient history, and identification of the scorpion species when available; and for the control group, the absence of any history of scorpion sting or exposure, no acute or chronic inflammatory condition, and willingness to participate.

Exclusion criteria: Exclusion criteria included pre-existing systemic diseases (e.g., autoimmune, renal, hepatic, or cardiac conditions); use of immunosuppressive or anti-inflammatory medications; and decline or withdrawal of informed consent. Blood samples were considered inadequate and excluded from analysis if there was evidence of hemolysis, insufficient serum volume for ELISA assays, or improper storage conditions prior to processing.

### 2.3 Clinical severity grading

Clinical severity was graded using a four-level system (Grade I–IV) based on local and systemic findings, adapted from the validated classification proposed by Isbister and Bawaskar.

Stage I: Mild pain or paresthesia at the bitten area.
Stage II: Very severe pain or paresthesia extending beyond the bitten area (entire extremity).
Stage III: Twitching, tremors, cranial nerve involvement in the extremities (eye movement disorder, blurred vision, dysphagia, salivation, tongue twitching and speech disorder, etc.).
Stage IV: Somatic neuromuscular, cranial nerve involvement. Along with pulmonary edema, myocardial infarction, shock, convulsions, etc.

### 2.4 Biochemical analysis

Venous blood samples were collected within the first few hours of hospital admission. After centrifugation at 3,000 rpm for 10 min, the serum samples were stored at −80 C until analysis. The following biomarkers were analyzed:

- SDF-4 (Stromal Cell-Derived Factor 4): Commercial ELISA kit (Catalog No: NBP2-75386, Novus Biologicals, USA).
- IL-13 (Interleukin-13): Human-specific ELISA kit (Catalog No: E-EL-H0104, Elabscience, USA).
- GPX4 (Glutathione Peroxidase 4): ELISA kit (Catalog No: EH8916, FN Test, China).

Additionally, thiol/disulfide homeostasis parameters were assessed using a fully automated spectrophotometric method based on dynamic thiol–disulfide exchange reactions. The following parameters were recorded: native thiol (µmol/L), total thiol (µmol/L), and disulfide (µmol/L). Ratios of disulfide/native thiol and disulfide/total thiol were calculated to assess oxidative stress balance.

### 2.5 Statistical analysis

All statistical evaluations were carried out using Jamovi statistical software (The jamovi project. (2024). jamovi (Version 2.6) (Computer software)). Prior to analysis, the distribution of continuous variables was assessed using the Shapiro–Wilk test. Non-normally distributed data were summarized using median values and interquartile ranges (IQR), while categorical variables were presented as frequencies and percentages.

Comparative analyses between patients with confirmed scorpion sting and those without were performed using the Mann–Whitney U test for skewed continuous variables, and the Pearson chi-square or Fisher’s exact test for categorical comparisons, depending on cell counts.

To explore the diagnostic utility of various biochemical markers, ROC curve analysis was conducted. The Youden Index was utilized to determine the most informative cut-off points. In addition to AUC (Area Under the Curve), key performance indicators such as sensitivity, specificity, PPV, and NPV were reported for each parameter.

Associations between biochemical profiles and the clinical severity grade of envenomation were examined using Spearman’s rank-order correlation. Moreover, binomial logistic regression was employed to identify variables independently associated with the presence of scorpion sting and to construct a predictive model. Regression results were reported with β coefficients, standard errors, odds ratios (OR) and 95% confidence intervals (CI), alongside p-values. Model adequacy and discrimination capacity were evaluated using McFadden’s pseudo-R^2^, Akaike Information Criterion (AIC), overall classification accuracy, and the model’s AUC from ROC analysis.

A significance threshold was set at p < 0.05 for all inferential procedures.

## 3 Results

A total of 60 patients with confirmed scorpion envenomation and 33 healthy individuals without envenomation were included in the study. Detailed clinical characteristics of the scorpion sting cases are presented in Table 1. The median age was 33.0 years (IQR: 25.0–44.0), with no statistically significant difference between groups regarding age or gender distribution (p > 0.05). Biochemical parameters revealed significant alterations in the scorpion sting group. Patients exhibited lower levels of native thiol (269.3 μmol/L vs. 351.7 μmol/L, p < 0.001) and total thiol (306.1 μmol/L vs. 404.4 μmol/L, p < 0.001), alongside elevated levels of disulfide (23.8 μmol/L vs. 12.7 μmol/L, p < 0.001). In the envenomated group, disulfide/native thiol and disulfide/total thiol ratios were significantly higher, while GPX4 levels were significantly lower compared to the control group (p < 0.001).

**TABLE 1:** Detailed clinical characteristics of scorpion sting cases.

Variable	Category	n = 60 (%) median (IQR)
Site of Sting	Hand/fingers	28 (47%)
	Foot/toes	7 (12%)
	Leg	4 (6.7%)
	Foot	11 (18%)
	Back	3 (5.0%)
	Head	4 (6.7%)
	Arm	3 (5.0%)
Scorpion Color	Unknown	4 (6.7%)
	Black	27 (45%)
	Yellow	29 (48%)
Pain	Yes	59 (98%)
Swelling	Yes	57 (95%)
Cold Extremities	Yes	19 (32%)
Hyperemia	Yes	58 (97%)
Burning Sensation	Yes	59 (98%)
Itching	Yes	51 (85%)
Nausea	Yes	17 (28%)
Sweating	Yes	17 (28%)
Hypotension	Yes	9 (15%)
Hypertension	Yes	21 (35%)
Dry Mouth	Yes	15 (25%)
Tachycardia	Yes	30 (50%)
Dyspnea	Yes	18 (30%)
Confusion	Yes	7 (12%)
Thirst	Yes	10 (17%)
Severity Grade	1	31 (50%)
	2	21 (35%)
	3	7 (12%)
	4	1 (1.7%)
Tongue Numbness	Yes	5 (8.3%)
Paresthesia	Yes	33 (55%)
Gender	Male	23 (38%)
	Female	37 (62%)
Education Level	No formal education	9 (15%)
	Primary school	23 (38%)
	High school	18 (30%)
	University	10 (17%)
Respiratory Rate	(breaths/min)	19 (16–22)

Additionally, serum levels of SDF-4, IL-13, and periostin were notably increased in the patient group (p < 0.001 for all, except IL-13: p = 0.019) (Table 2).

**TABLE 2:** Comparison of biochemical and clinical parameters according to scorpion sting.

Variable	Scorpion sting n = 60, median (IQR)	Control n = 33, median (IQR)	Total median (IQR)	p-value
Native Thiol (µmol/L)	269.3 (241.1–291.6)	351.7 (298.3–397.7)	288.6 (252.3–343.9)	<0.001
Total Thiol (µmol/L)	306.1 (281.8–333.2)	404.4 (362.6–450.1)	329.4 (292.1–395.6)	<0.001
Disulfide (µmol/L)	23.8 (18.9–26.8)	12.7 (6.5–14.3)	18.4 (13.1–24.6)	<0.001
Disulfide / Native Thiol (%)	7.6 (6.0–10.5)	4.3 (3.2–5.0)	6.7 (4.4–8.3)	<0.001
Disulfide / Total Thiol (%)	6.7 (5.2–8.7)	3.9 (3.0–4.5)	5.8 (4.1–7.9)	<0.001
Native Thiol / Total Thiol (%)	86.9 (82.8–89.5)	92.1 (90.9–94.0)	88.4 (85.8–92.0)	<0.001
SDF-4 (pg/mL)	311.1 (213.4–386.1)	119.2 (116.6–121.0)	198.5 (119.2–351.0)	<0.001
IL-13 (pg/mL)	147.2 (48.7–237.0)	90.8 (84.3–125.0)	125.0 (74.6–169.2)	0.019
GPX4 (ng/mL)	420.7 (407.7–432.3)	314.6 (311.0–325.7)	407.3 (320.8–426.6)	<0.001
Periostin (ng/mL)	5.4 (4.3–8.7)	3.6 (3.1–4.2)	4.5 (3.5–6.1)	<0.001
BMI (kg/m^2^)	23.8 (23.5–24.4)	24.1 (23.6–24.5)	23.9 (23.6–24.4)	0.286
Age (years)	33.0 (24.8–45.0)	33.0 (26.0–43.0)	33.0 (25.0–44.0)	0.888
Gender (Female, %)	23 (38.3%) F	13 (39.4%) F	36 (38.7%) F	1.000
Education Level (categorical)	0: 15.0% 1: 38.3% 2: 30.0% 3: 16.7%	0: 18.2% 1: 33.3% 2: 30.3% 3: 18.2%	0: 16.1% 1: 36.6% 2: 30.1% 3: 17.2%	0.958
Residence (categorical)	1: 48.3% 2: 15.0% 3: 36.7%	1: 45.5% 2: 12.1% 3: 42.4%	1: 47.3% 2: 14.0% 3: 38.7%	0.841
Systolic BP (mmHg)	129.0 (117.8–140.2)	120.0 (118.0–120.0)	120.0 (118.0–137.0)	0.009
Diastolic BP (mmHg)	84.0 (70.0–92.2)	80.0 (70.0–87.0)	80.0 (70.0–90.0)	0.403
Pulse (beats/min)	95.0 (78.8–109.0)	84.0 (78.0–89.0)	88.0 (78.0–99.0)	0.002

Abbreviations: BMI, body mass index; SDF-4, Stromal Cell-Derived Factor 4; IL-13, Interleukin-13; GPX4, Glutathione Peroxidase 4; Periostin, Periostin protein; BP, blood pressure.

Education Level Categories: 0, No formal education; 1, Primary school; 2, High school; 3, University.

Residence: 1, Rural; 2, Semi-urban; 3, Urban.

**FIGURE 1:**
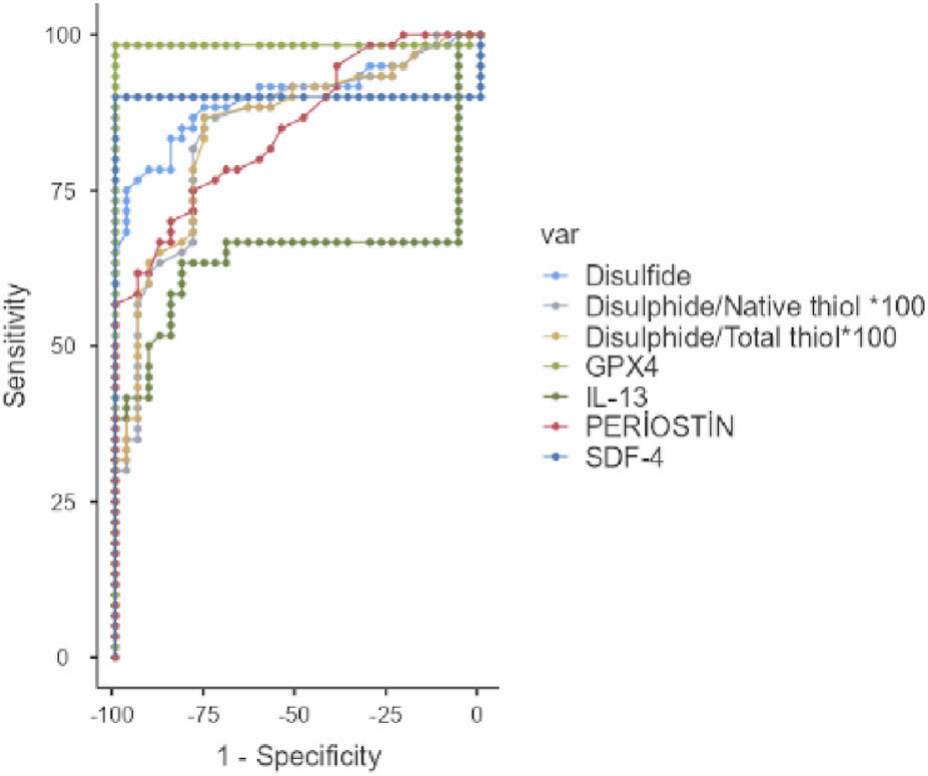
ROC curves of selected biochemical parameters for predicting scorpion sting cases. Abbreviations: SDF-4, Stromal Cell-Derived Factor 4; IL-13, Interleukin-13; GPX4, Glutathione Peroxidase; Var, variable.

**FIGURE 2:**
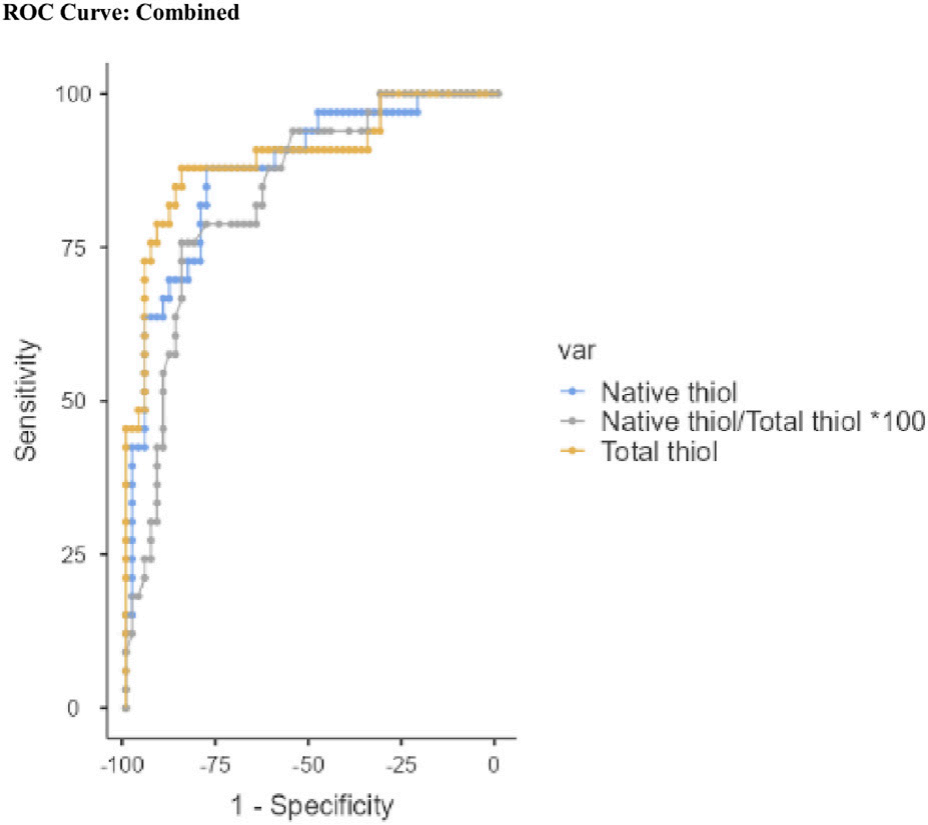
ROC curves of selected biochemical parameters for predicting the absence of scorpion sting. Abbreviations: AUC, Area Under the Curve. Var, variable.

ROC curve analysis demonstrated high diagnostic accuracy for several parameters. GPX4 (AUC = 0.984), SDF-4 (AUC = 0.900), periostin (AUC = 0.850), and the disulfide/native thiol ratio (AUC = 0.845) were the most reliable predictors for distinguishing envenomated patients from controls. Youden index-based optimal cut-off values further supported their predictive utility (Table 3; Figure 1). Notably, GPX4 had a sensitivity and specificity of 100% and 93.3%, respectively. Parameters with high negative predictive value included native thiol (AUC = 0.876) and total thiol (AUC = 0.898), which were effective for excluding scorpion sting when above their respective thresholds (Table 4; Figure 2).

**TABLE 3:** Predictive performance of biochemical parameters in scorpion sting diagnosis based on ROC analysis.

Parameter	Cut-off	Sensitivity (%)	Specificity (%)	PPV (%)	NPV (%)	Youden index	AUC
Disulfide (µmol/L)	19.0	75.0	96.97	97.83	68.09	0.72	0.900
Disulfide / Native Thiol (%)	5.5	86.67	75.76	86.67	75.76	0.624	0.845
Disulfide / Total Thiol (%)	4.7	86.67	75.76	86.67	75.76	0.624	0.847
SDF-4 (pg/mL)	128.3	90.0	100.0	100.0	84.62	0.9	0.900
IL-13 (pg/mL)	136.9	63.33	81.82	86.36	55.1	0.452	0.647
GPX4 (ng/mL)	394.9	98.33	100.0	100.0	97.06	0.983	0.984
Periostin (ng/mL)	5.3	56.67	100.0	100.0	55.93	0.567	0.850

Abbreviations: SDF-4, Stromal Cell-Derived Factor 4; IL-13, Interleukin-13; GPX4, Glutathione Peroxidase 4; Periostin, Periostin protein; AUC, area under the curve; PPV, positive predictive value; NPV, negative predictive value.

**TABLE 4:** ROC analysis for predicting absence of scorpion sting.

Parameter	Cut-off	Sensitivity (%)	Specificity (%)	PPV (%)	NPV (%)	Youden index	AUC
Native Thiol (µmol/L)	295.1	87.88	78.33	69.05	92.16	0.662	0.876
Total Thiol (µmol/L)	340.3	87.88	85.0	76.32	92.73	0.729	0.898
Native Thiol / Total Thiol (%)	90.9	75.76	85.0	73.53	86.44	0.608	0.835

Abbreviations: AUC, area under the curve; PPV, positive predictive value; NPV, negative predictive value.

Correlation analysis between biochemical markers and clinical severity grade revealed significant associations. Disulfide (r = 0.515, p < 0.001), disulfide/native thiol (r = 0.653, p < 0.001), disulfide/total thiol (r = 0.632, p < 0.001), and periostin (r = 0.495, p < 0.001) demonstrated positive correlations with clinical severity. Conversely, native thiol (r = −0.161, p = 0.223) and GPX4 (r = −0.109, p = 0.407) showed weak negative correlations, but these were not statistically significant (Table 5).

**TABLE 5:** Spearman’s rank correlation coefficients (rho) between clinical severity grade and selected biochemical parameters, along with corresponding p-values.

Biochemical parameter	Spearman’s ρ with grade	p-value
Native Thiol (µmol/L)	−0.161	0.219
Total Thiol (µmol/L)	0.038	0.770
Disulfide (µmol/L)	0.515	<0.001
Disulfide / Native Thiol (%)	0.653	<0.001
Disulfide / Total Thiol (%)	0.632	<0.001
Native Thiol / Total Thiol (%)	−0.640	<0.001
SDF-4 (pg/mL)	0.196	0.134
IL-13 (pg/mL)	0.381	0.003
GPX4 (ng/mL)	−0.109	0.407
Periostin (ng/mL)	0.495	<0.001

Abbreviations: SDF-4, Stromal Cell-Derived Factor 4; IL-13, Interleukin-13; GPX4, Glutathione Peroxidase 4; Periostin, Periostin protein; ρ, Spearman correlation coefficient.

When patients were divided based on the presence of confusion (n = 7 vs. n = 53), significantly lower GPX4 and thiol levels and higher disulfide, IL-13, periostin, and SDF-4 levels were observed in the confused group (p < 0.01 for all comparisons) (Table 6).

**TABLE 6:** Comparison of biochemical parameters according to confusion Status.

Parameter	Confused = No n = 53 (median, IQR)	Confused = Yes n = 7 (median, IQR)	Total (median, IQR)	p-value
Native Thiol (µmol/L)	272.1 (246.7–293.8)	236.8 (225.3–247.7)	269.3 (241.1–291.6)	0.046
Disulfide (µmol/L)	23.3 (18.2–25.7)	29.8 (27.6–33.5)	23.8 (18.9–26.8)	0.003
Disulfide / Native Thiol (%)	7.4 (5.8–9.3)	12.0 (11.8–13.5)	7.6 (6.0–10.5)	<0.001
Disulfide / Total Thiol (%)	6.5 (5.2–8.0)	10.6 (9.6–10.7)	6.7 (5.2–8.7)	<0.001
Native Thiol / Total Thiol (%)	87.1 (84.3–89.7)	80.6 (78.7–80.9)	86.9 (82.8–89.5)	<0.001
IL-13 (pg/mL)	143.8 (26.9–194.4)	299.5 (260.9–391.8)	147.2 (48.7–237.0)	0.001
Periostin (ng/mL)	5.3 (4.1–6.7)	21.2 (20.4–22.2)	5.4 (4.3–8.7)	<0.001

Abbreviations: IL-13, Interleukin-13; Periostin, Periostin protein; IQR, interquartile range; µmol/L, micromoles per liter; ng/mL, nanograms per milliliter; pg/mL, picograms per milliliter.

**FIGURE 3:**
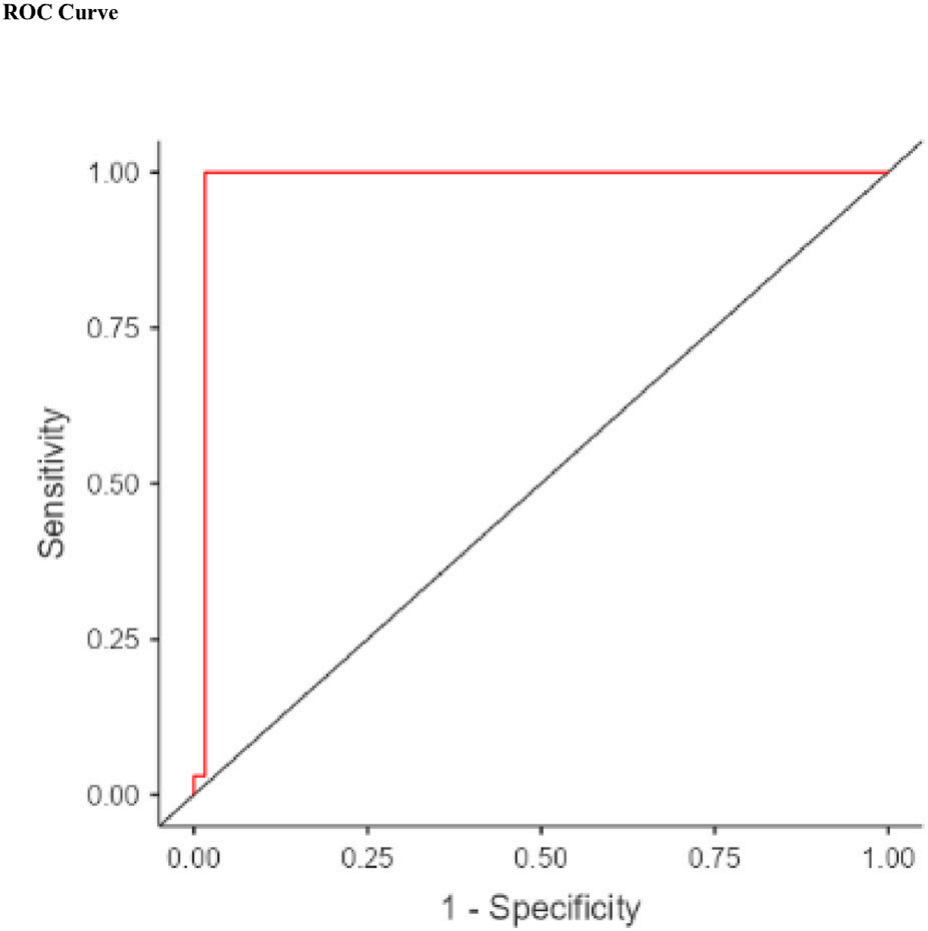
ROC curve demonstrating the diagnostic performance of the scorpion sting prediction model.

Binomial logistic regression analysis identified disulfide (β = −0.2075, p = 0.030) and GPX4 (β = −0.0590, p < 0.001) as independent predictors of scorpion envenomation. Despite higher disulfide levels in patients, the negative β in the logistic model likely reflects the binary coding structure (1 = control, 0 = envenomated), where increasing disulfide values decrease the odds of being in the control group. The regression model demonstrated excellent fit and performance, with a chi-square value of 98.6 (p < 0.001), McFadden’s R^2^ of 0.815, and an AIC of 28.4. The model correctly classified 98.9% of cases, with a sensitivity of 1.00 and specificity of 0.983 (Table 7). The corresponding ROC curve illustrated the model’s high discriminative power with an AUC of 0.984 (Table 8; Figure 3).

**TABLE 7:** Logistic regression model for predicting scorpion sting and clinical severity model coefficients.

Predictor	Estimate (β)	Standard error (SE)	Z	p-value	Odds ratio (OR)	95% CI (lower)	95% CI (Upper)
Disulfide (µmol/L)	−0.2075	0.0957	−2.17	0.030	0.813	0.674	0.980
GPX4 (ng/mL)	−0.059	0.0137	−4.29	<0.001	0.943	0.918	0.968

Abbreviations: OR, odds ratio; CI, confidence interval; AIC, akaike information criterion; SE, standard error; GPX4, Glutathione Peroxidase 4; µmol/L, micromoles per liter; ng/mL, nanograms per milliliter.

**TABLE 8:** Logistic regression model for predicting scorpion sting and clinical severity model performance metrics.

Metric	Value
Model Chi-square (χ^2^)	98.6
Degrees of Freedom	2
p-value	<0.001
R^2^ (McFadden)	0.815
AIC	28.4
Accuracy	0.989
Sensitivity	1.00
Specificity	0.983
AUC	0.984

Abbreviations: AIC, akaike information criterion; AUC, area under the curve.

## 4 Discussion

This study is the first to evaluate the diagnostic value of IL-13, periostin, SDF-4, GPX4, and thiol/disulfide homeostasis parameters in adult patients with scorpion envenomation. Our findings indicate that GPX4 levels significantly decreased, while IL-13, periostin, and SDF-4 levels increased in the envenomated group, accompanied by marked disruption in thiol/disulfide balance. These results reveal distinct systemic biochemical responses reflecting oxidative stress and inflammation in scorpionism.

Oxidative stress, resulting from an imbalance between ROS production and antioxidant defenses, plays a central role in the pathophysiology of envenomation-induced tissue damage (Petricevich, 2010). We observed significant reductions in native and total thiol levels, with corresponding increases in disulfide and disulfide/thiol ratios, supporting previous findings in inflammatory conditions such as sepsis and gestational diabetes (Erel and Neselioglu, 2014; Aydin et al., 2020; Cindoglu et al., 2023). These shifts reflect the consumption of antioxidant reserves and suggest a compensatory redox mechanism that, when overwhelmed, may lead to progressive cellular injury.

Another key finding was the significant decline in GPX4 levels among patients. GPX4 is a crucial antioxidant enzyme that protects cells from ferroptosis via inhibition of lipid peroxidation (Friedmann Angeli et al., 2014; Hu et al., 2025). Its depletion has been associated with tubular necrosis and mortality in experimental models of oxidative injury (Brigelius-Flohé and Maiorino, 2020). Thus, reduced GPX4 in our patients may represent both oxidative stress burden and impaired cellular defense during envenomation.

Periostin, strongly induced by IL-13 and IL-4, is known to mediate tissue remodeling and fibrosis in chronic inflammation (Masuoka et al., 2012; Conway et al., 2014; Wynn, 2003). Elevated periostin levels in our study suggest active tissue response to venom-induced inflammation and injury, potentially reflecting both damage and repair mechanisms. The positive correlation between periostin and clinical severity score further highlights its role as a prognostic indicator.

Similarly, IL-13 levels were significantly higher in patients and were associated with disease severity. As a central Th2 cytokine, IL-13 contributes to ROS generation, mucus hypersecretion, and fibrosis in multiple models (Wynn, 2003; Zhu et al., 1999; Gieseck et al., 2021). Its elevation in severe envenomation cases may reflect broader systemic immune activation, supporting its potential as a marker of severity.

SDF-4, a Golgi-associated protein involved in cellular stress responses, also showed significant elevation in patients. Prior studies have shown SDF-4 upregulation under endoplasmic reticulum stress and its protective role in maintaining cellular homeostasis (Chen et al., 2014; Luo et al., 2016). Its increase in our patients likely represents a cellular adaptation to venom-induced stress and suggests its emerging value as a diagnostic biomarker.

Importantly, logistic regression analysis identified disulfide and GPX4 as independent predictors of envenomation, and ROC analyses demonstrated high diagnostic performance, particularly for GPX4 (AUC = 0.984), SDF-4 (AUC = 0.900), and periostin (AUC = 0.850). These findings suggest these markers not only reflect underlying pathophysiology but may also aid early diagnosis and risk stratification.

The identification of GPX4 and disulfide/native thiol ratio as independent predictors suggests that these biomarkers may serve as rapid laboratory indicators of envenomation severity in emergency settings. Their integration into initial biochemical panels could help differentiate mild from severe cases and guide timely interventions such as hospital admission, cardiac monitoring, or antivenom administration. Furthermore, periostin and IL-13 may serve as adjunctive markers for systemic inflammation and tissue injury, aiding in the risk stratification of patients presenting with ambiguous clinical findings.

In conclusion, this study identifies GPX4, disulfide/native thiol ratio, IL-13, and periostin as clinically relevant biomarkers that can improve diagnostic precision and risk stratification in adult scorpion envenomation. Their incorporation into emergency biochemical assessment protocols could provide a rapid, objective complement to clinical examination, enhancing early triage and management decisions.

Strengths of the study: This is the first study to jointly assess GPX4, IL-13, periostin, SDF-4, and thiol/disulfide homeostasis in adult scorpion envenomation, addressing a gap where most data come from pediatric or purely clinical studies. The prospective design and inclusion of age- and sex-matched healthy controls enhance reliability by reducing bias and ensuring standardized biomarker measurement. Simultaneous assessment of antioxidant, inflammatory, remodeling, and stress-related markers provides a multidimensional view of the oxidative and inflammatory response. Linking biomarker changes to severity grades demonstrates their potential for early risk stratification and emergency management. The results underscore the role of oxidative stress and inflammation in scorpion envenomation and suggest targets for future diagnostic and therapeutic strategies.

## 5 Limitations

This study has several limitations that should be acknowledged. First, the sample size was relatively small and derived from a single tertiary care center, which may limit the generalizability of the findings to broader populations and other geographical regions. Second, the study lacked longitudinal follow-up data to determine the prognostic value of the biomarkers in long-term outcomes or delayed complications. Third, although efforts were made to exclude patients with underlying systemic diseases, subclinical inflammatory or oxidative conditions might have confounded some of the biomarker measurements. Fourth, Although the study was conducted in a region where Androctonus crassicauda is the predominant species, the oxidative and inflammatory pathways involved are largely conserved across scorpion species. Nevertheless, external validation in populations exposed to different species and venom profiles is warranted before generalizing these findings. Additionally, the use of ELISA kits from different manufacturers may introduce inter-assay variability, despite standardized laboratory procedures. Finally, the high AUC values observed in the ROC analysis, particularly for GPX4, may reflect potential overfitting due to the lack of external validation or an independent replication cohort.

## Data Availability

The original contributions presented in the study are included in the article/supplementary material, further inquiries can be directed to the corresponding author.
